# Quantitative spatial evaluation of tumor-immune interactions in the immunotherapy setting of metastatic melanoma lymph nodes

**DOI:** 10.3389/fimmu.2022.1024039

**Published:** 2022-12-05

**Authors:** Rachel L. G. Maus, Alexey A. Leontovich, Raymond M. Moore, Zachary Fogarty, Ruifeng Guo, Tara M. Davidson, Burak Tekin, Chathu Atherton, Jill M. Schimke, Betty A. Dicke, Benjamin J. Chen, Svetomir N. Markovic

**Affiliations:** ^1^ Department of Oncology, Mayo Clinic, Rochester, MN, United States; ^2^ Department of Computational Biology, Mayo Clinic, Rochester, MN, United States; ^3^ Department of Anatomic Pathology, Mayo Clinic, Rochester, MN, United States; ^4^ Department of Internal Medicine, Mayo Clinic, Rochester, MN, United States; ^5^ Department of Translational Research Pathology, Bristol Myers Squibb, Cambridge, MA, United States

**Keywords:** tumor microenvironment, multiplex immunofluorescence, melanoma, immunotherapy, spatial biology

## Abstract

**Introduction:**

Immune cell infiltration into the tumor microenvironment is generally associated with favorable clinical outcomes in solid tumors. However, the dynamic interplay among distinct immune cell subsets within the tumor-immune microenvironment as it relates to clinical responses to immunotherapy remains unresolved. In this study, we applied multiplex immunofluorescence (MxIF) to spatially characterize tumor-immune interactions within the metastatic melanoma lymph node.

**Methods:**

Pretreatment, whole lymph node biopsies were evaluated from 25 patients with regionally metastatic melanoma who underwent subsequent anti-PD1 therapy. Cyclic MxIF was applied to quantitatively and spatially assess expression of 45 pathologist-validated antibodies on a single tissue section. Pixel-based single cell segmentation and a supervised classifier approach resolved 10 distinct tumor, stromal and immune cell phenotypes and functional expression of PD1.

**Results:**

Single cell analysis across 416 pathologist-annotated tumor core regions of interest yielded 5.5 million cells for spatial evaluation. Cellular composition of tumor and immune cell subsets did not differ in the tumor core with regards to recurrence outcomes (p>0.05) however spatial patterns significantly differed in regional and paracrine neighborhood evaluations. Specifically, a regional community cluster comprised of primarily tumor and dendritic cells was enriched in patients that did not experience recurrence (p=0.009). By an independent spatial approach, cell-centric neighborhood analyses identified an enrichment for dendritic cells in cytotoxic T cell (CTL) and tumor cell-centric neighborhoods in the no recurrence patient response group (p<0.0001). Further evaluation of these neighborhoods identified an enrichment for CTL-dendritic cell interactions in patients that did not experience recurrence (p<0.0001) whereas CTL-macrophage interactions were more prevalent in CTL-centric neighborhoods of patients who experienced recurrence (p<0.0001).

**Discussion:**

Overall, this study offers a more comprehensive evaluation of immune infiltrates and spatial-immune signatures in the metastatic tumor-immune microenvironment as it informs recurrence risk following immunotherapy.

## Introduction

The success of immunotherapy is intrinsically related to the surrounding tumor-immune microenvironment (TIME). To assess the degree of immune infiltration in tumor tissue, the density of tumor infiltrating lymphocytes (TILs) has emerged as a relevant prognostic biomarker in the primary-tumor setting of solid tumors including melanoma (1–5). While an increased density of TILs has been largely associated with favorable prognosis in melanoma (4), durable clinical responses to immune checkpoint inhibitors are only achieved in a small subset of patients (6, 7). Therefore, comprehensive detailing of the immune infiltrate composition, the spatial arrangement of immune cells within the TIME and the cellular interactions promoted within these spatial structures is essential to improve patient stratification to immunotherapy regimens.

Current immune checkpoint inhibitors mechanistically target the cytotoxic T cell (CTL)-tumor cell interaction (8). CTLs are effector lymphocytes capable of mounting an anti-tumor immune response. To enable CTL-mediated tumor cell lysis, CTLs must first be primed by an antigen presenting cell (macrophage, dendritic cell, B cell) and positioned near the target tumor cell. Increasing evidence suggests this effector-target mechanism takes place in a diverse tumor-immune climate comprised of tumor-associated macrophages, myeloid-derived suppressor cells, and regulatory T cells with a propensity to promote a tolerogenic TIME; while CTLs, Th1 biased helper T cells and dendritic cells (DCs) may offer a counterbalance in favor of anti-tumor immune responses (9–11). Resolving the paracrine interactions within this diverse tumor-immune climate may reveal yet unrealized interactions capable of predicting the success of immune checkpoint inhibitors in increasingly complex microenvironments.

Recent advances in bioimaging platforms have enabled highly multiplexed, single-cell microscopy to be applied to clinically archived formalin-fixed paraffin embedded (FFPE) tissue specimens (12). By light microscopy or mass-spectrometry based methodologies, these multiplexed platforms have been applied to tissue microarrays to quantitatively assess the spatial distribution and phenotypic diversity of the cellular landscape in various cancers including colorectal (13, 14), ovarian (15), and breast cancer (16–19). Recently, we expanded this application to include pathologist-guided sampling of distinct histologies within the metastatic melanoma lymph node (LN) (20). Together these platforms have enabled studies on the tumor-immune landscape within tissue and are beginning to cast a light on the cellular diversity and spatial organization of the TIME as it informs therapeutic responses.

TILs are currently used as a surrogate for responsiveness to immunotherapy (“hot” vs “cold” tumors) (21). But such work does not address the diversity and functional status of TILs within the spatial setting of the metastatic tumor and fails to consider other contributing factors present in the TIME which create the overall immune contexture of the tumor. Therefore, to see whether TIL diversity and spatial interactions within the TIME inform clinical responsiveness to anti-PD1 therapy, we studied differences in metastatic LN tissues from patients with melanoma prior to treatment with anti-PD1 immunotherapy.

## Materials and methods

### Patient demographics

From an initial cohort of thirty-three treatment-naïve patients with Stage III melanoma eligible for anti-PD1 immunotherapy, twenty-five patients were selected for MxIF analysis in the current study due to having sufficient tumor regions in the LN imaged to satisfy the tumor core field of view (FOV) criteria. Use of all patient biospecimens and clinical data was collected in accordance with the Declaration of Helsinki with approval obtained by Mayo Clinic’s Institutional Review Board (IRB). Given the retrospective, minimal risk structure of the study design, informed written consent from participants was waived by the IRB. Research authorization was verified for each patient prior to use of samples in the current study.

### Tissue preparation and antigen retrieval

A single 5µm formalin-fixed paraffin embedded (FFPE) tissue section was prepared from each patient for the MxIF workflow. Slide preparation including deparaffinization, rehydration and permeabilization was performed on each slide as detailed previously (20). Heat-induced epitope retrieval was performed in a NxGen decloaking chamber (Biocare Medical) using heated citrate buffer (pH 6, Vector Labs) and Tris-EDTA buffer (pH 9.5). Slides were then blocked in 10% donkey serum, stained in DAPI (1ug/mL) and coverslipped using non-hardening mounting media.

### Antibody panel design

We customized a 45-antibody panel to characterize tumor, immune and stromal cell subsets into 10 unique classification phenotypes. Antibody clone information is detailed in **Supplemental Table 1**. Antibodies were purified on HiTrap Protein A or Protein G columns (Sigma) or obtained as carrier-free formulations. Direct fluorophore conjugation was performed using Bis NHS Ester dye (GE Healthcare) as previously described (13).

Similar to previous work (20), MxIF antibodies were evaluated by an experienced board-certified dermatopathologist and compared to routine chromogenic IHC stains to ensure concordance. When unavailable, corresponding H&E sections were reviewed to determine cell distributions in distinct cell types. MxIF antibodies also underwent random quality checking to ensure reproducibility following an antibody lot change.

### Field of view selection

Images were acquired by the INCell Analyzer 2500HS (GE Healthcare) beginning with a whole slide image of the LN tissue at the 10X objective using DAPI and Cy3 fluorescent channels and was projected as a virtual H&E image for pathology review. Together with a serial H&E section, histopathological regions of interest were selected by a board-certified pathologist encompassing areas of tumor core, tumor-immune interface and lymphoid tissue. Individual fields of view (2040 x 2040 pixels, ~1mm^2^) were then selected from these histopathological regions at the 20X objective and used for all downstream multiplexed imaging (**Supplemental Figure 1**). FOVs localized to the tumor core were identified in 25 cases of the initial cohort and used for the current study (n=416 FOVs).

### Image acquisition

All images were acquired by the INCell Analyzer 2500HS (GE Healthcare). Following background and FOV selection at the 10X and 20X objectives, user-annotated FOVs were imaged iteratively at the 20X objective. Details regarding each image type has been detailed previously (20). The autofluorescence (AF) removed image encompasses the final stained image following per-pixel image subtraction of the dye inactivation image from the previous round. Image normalization was applied to scale the intensities according to the minimum exposure time in the dataset. Image alignment was performed for each round and compared to the initial 10X DAPI image using Insight Toolkit registration and phase correlation.

### Cyclic MxIF process

Evaluation of multiple antibodies on a single tissue section was achieved through cyclic MxIF methods including iterative rounds of fluorescent antibody staining and dye inactivation. For staining, slides were incubated for 1 hour at RT in a humidified dark chamber. Slides were washed in PBS and coverslipped for imaging. Following imaging, slides were de-coverslipped in PBS and subjected to dye inactivation in a sodium bicarbonate solution (0.5M NaHCO_3_, pH 11.2) as previously described (13). Following dye inactivation, slides were washed in PBS and coverslipped for imaging.

### Pixel classification-based segmentation

We adapted the pixel classification process developed previously by the Bodenmiller lab to generate boundaries around each cell and nucleus compartment (18). The separate TIFF image files from the INCell were joined into a standard OME.TIFF file format, readable by BioFormats (22). The process includes encoding individual pixels into one of three classes to differentiate nuclear, cytoplasm and background areas using the Ilastik package, resulting in three probability prediction matrices (23). We enhanced our pixel classification model by employing 14 common core biomarkers (DAPI, CD14, CD163, CD16, CD206, CD20, CD45, CD4, CD68, CD8, gp100, HLA-II, HLA-I, NaKATPase, S6) to leverage pixel classification from previous projects of similar tissue (20). Supervised m odel training was conducted by labe ling a tiled grid of crops (200x200 pixels) from each FOV in the dataset. The single pixel uncertainty was calculated and evaluated on labe led and unlabeled crops to determine the ability for algorithmic detection to distinguish between classes. From this effort we expanded on the existing pixel classification model from prior work (20), thus obtained 12.40% +/- 0.5% uncertainty in 140 validation crops. The updated model predictions were then applied to every FOV in the dataset generating pixel classification probabilities which were then input into CellProfiler for algorithmically constrained object propagation and the generation of labe led masks for whole cell and nuclei (24). These segmentation masks were merged with the full panel OME.TIFF MxIF image files and incorporated into QuPath, an open-source software package for quantitative bioimaging analysis (25). Using a QuPath script to merge disparate data files, QuPath was then used to export all quantifications.

### Classification and single cell analysis

Cell classification was divided into two separate approaches for distinct objectives : phenotypic and functional classification.

Phenotypic classification took a supervised classifier approach which predicted the cellular phenotype for the ~1.4 million (1,445,062) cells located in FOVs labe led as ‘tumor core’. After several model testing iterations [SVM, GBM, RF, ANN] and parameters, the best supervised classification approach converged on two models leveraging the hierarchical nature of cell classification. The first model predicted 3 broad classes of tumor, stroma and immune cells. This was followed by a second model where both the tumor and stromal classes were retained and higher-granularity classification was provided to the immune cell class. Expert cell annotations were provided, amassing a training dataset of 169,101 cell labels [Tumor Cell, Macrophage, Dendritic Cell, Stromal Cell, B Cell, Cytotoxic T Cell, Helper T Cell, Neutrophil, Unclassified Immune, Regulatory T Cell, and Monocyte]. For annotation, cells were assigned to a single class by visualizing FOVs in QuPath and leveraging expression of 18 antibodies from the panel design. The phenotypic classification assignment schema is summarized in **Supplemental Table 2**.

In both instances of model training, the datasets were split into 2/3 for training and 1/3 testing. . During model development and training, various modeling approaches were attempted, thus the top performing approach was a Gradient Booster Classifier (GBM) for the first model and a SVM for the second (26). The first GBM model parameterized by 500 estimators, max depth of 4, and a learning rate of 0.25. The resulting first GBM classifier obtained a training accuracy of 96.61% and a test accuracy of 92.63% across 3 classes. The second SVM classifier obtained a training accuracy of 75.54% and a test accuracy of 69.41% across 9 classes.

For functional classification of PD1, a gating strategy was preferred, in pursuit of a more specific binary classification. To remove noisy artifacts and normalize a single signal to a binary threshold more aggressively, an arcsnih transformation was employed to reduce the spectrum of values from 16-bit quantification. Since PD1 was known to represent a minor fraction of cells in the entire dataset, first the peak of each distribution per collection of FOVs belonging to the same biospecimen was identified, then moved to zero. StandardScaler was used to scale the entire dataset (26). The resulting distribution was then threshold on two axes, cell quantification mean and standard deviation. The gating values for these thresholds were identified as the value representing the fixed false positive rate, between a small representative set of annotations provided, by multiple annotators and visually validated.

Classification labels for both cellular phenotypes and expression of functional markers were returned to QuPath and merged with the spatial coordinates of each cell, its segmentation profile and classification label to visualize and quantify single metrics in all downstream analytics. This per-cell assignment was then mapped back to the FOV and compared to the original MxIF overlay for visual assessment and validation.

### Cellular communities

Originally referred to as “Neighbor Coordination” by the originators (14), we shifted the terminology to “Cellular Community” to distinguish this from the term neighborhoods, as they are derived separately and distinctly. The term community refers to the diversity of proportions of cell types which are spatially arranged around each single cell, thus creating a relational structure to define a cell’s environment. Wherein, that relationship is established between cells, based on some number of nearest cells. Two parameters are required to implement the original method as provided, which are; the N number of cells to aggregate *via* sklearn.neighbors method NearestNeighbors, and c number of clusters to result from the sklearn.cluster method MiniBatchKMeans (14, 26), Strategy in optimal parameter selection was not provided, however the code does allow for efficient prototyping. We reasoned the number of community clusters should be reflective of the number of phenotypic cell classes + 1 resulting in c=10. We further reasoned that the number of cells comprising a community should represent tumor microenvironment including distinct tumor-immune interactions. Community sizes were assessed at various sizes k=5,25,50,75,100, with k=75 resulting in distinct macro-architectures within the tumor tissue ((i.e. primarily tumor-dominant communities vs tumor communities with either lymphocyte or myeloid dominant immune cell subsets).

### Index cell-centric cellular neighborhoods

Analysis of the TIME was performed using SpatStat package in R programming language, using cell neighborhood analysis (CNA) algorithm (27). This algorithm uses SpatStat function to traverse through every point on a 2-dimensional plane and create a neighborhood of a requested size (57 pixel diameter). CNA then counts cells of every class in each neighborhood and records coordinates and counts in a matrix. In the current study, neighborhoods were created around each tumor cell and CTL, named tumor centric cellular neighborhood (TCCN) and cytotoxic T cell-centric cellular neighborhood (CTCN) respectively. Derivation of the size of the CN for this study is summarized in **Supplemental Figure 2**.

### Statistical analyses

Statistical tests were performed on a per-patient basis. Due to the difference in population size between the two response groups, the non-parametric Pearson’s chi-squared correlation statistic was used to compare differences between patients that experienced melanoma recurrence from those that did not. The adjusted p-values less than 0.05 were reported as significant.

## Results

### Patient population

The regional LN remains the most common first site of metastatic disease in patients with melanoma (28). To intervene and assess the extent of spread, patients presenting with Stage IB or II melanoma may undergo surgical resection of the LN prior to receiving immunotherapy regimens. From an initial cohort of 33 MM patients, 25 were identified as having 1) whole excisional LN biopsy material; 2) sufficient regions (FOVs) in the tumor core available; and 3) underwent a similar treatment course receiving anti-PD1 therapy with adjuvant single-agent nivolumab (either 240 mg every 2 weeks or 480mg every 4 weeks). Patient demographics and recurrence status for the final cohort of 25 patients following anti-PD1 therapy is summarized in **Supplemental Table 1**. Nine patients experienced recurrence in 12 months of less, similar to recurrence rates in previous reports (29). The two outcome groups did not statistically differ in terms of gender, age, tumor characteristics at baseline or average follow-up in days after nivolumab treatment. Median follow-up from last day of anti-PD1 therapy to death or last patient contact for the entire cohort was 30.2 months. Four patients in the relapsed < 1 year cohort (recurrence group) died related to disease, and no deaths were reported in the cohort of patients who did not experience relapse (>1 year) (no recurrence group).

### Cyclic MxIF applied to resected LN biopsies enables classification and quantification of the TIME in the melanoma tumor core

**Figure 1:**
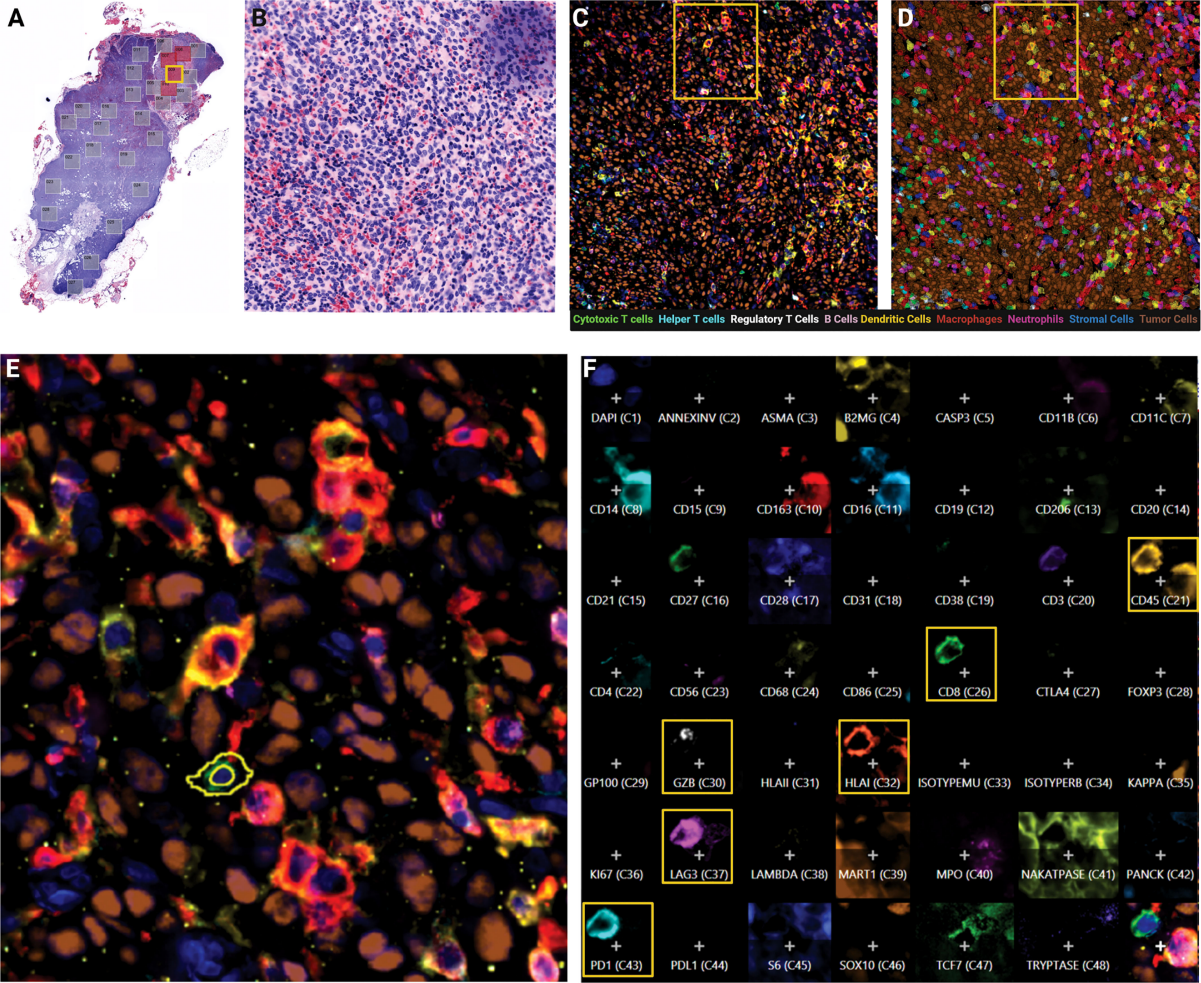
Cylic MxIF applied to whole excisional lymph node biopsies provides phenotypic and functional status of single cells in the tumor core. **(A)** Virtual H&E of whole excisional LN biopsy at 10X objective with pathologist-annotated tumor mass fields of view indicated (red boxes). **(B)** Virtual H&E of single field of view (FOV) at 20X objective. **(C)** MxIF overlay of the same FOV visualizing 9 cellular phenotypes with 12 antibodies. **(D)** Phenotypic classification overlay, filled colors indicate assigned cell class. **(E)** Inset from yellow outline in panel **(D)** indicating expression profile of individual cells. **(F)** Composite expression profile of all markers in the panel design for a single cell highlighted in Panel **(E)**.

By H&E clinical evaluation, TILs are assessed for their presence or absence in the primary tumor but rarely further differentiated or evaluated in the metastatic setting. In this study, we sub-selected pathologist annotated tumor core regions (n=416 FOVs) within metastatic LN resected tissue to delineate the tumor, immune and stromal climate within this specific microenvironment (**Figures 1A, B**). Utilizing cyclic MxIF technology, the tumor core spatial landscape was differentiated at increasing levels of resolution including the tissue macroarchitecture and the phenotypic and functional status of diverse cell subsets. Leveraging the expression of 45 biomarkers on a single tissue section, the cellular landscape was visually assessed, phenotypically and functionally classified and quantitatively measured (**Figures 1C, D**). The high precision enabled at the 20X objective further enhanced the resolution at which subcellular localization of biomarker expression was visually assessed and quantitatively captured (**Figures 1E, F**).

### Patient level differences in cell composition

**Figure 2:**
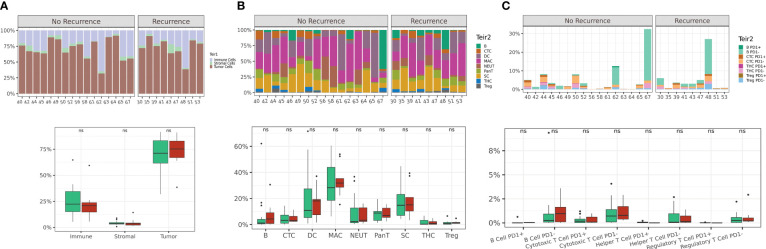
Single cell quantification of tumor core regions is not sufficient to differentiate based on recurrence outcome. Abundance plots of 3-tier classification (tumor, immune and stromal cells) across all FOVs on a per-patient basis **(A)**. Abundance plots of immune cell phenotypic classes **(B)** and abundance plots of TIL subsets based on PD1 expression **(C)**. Distribution of patients displayed on box plot as green circles (no recurrence) and red triangles (recurrence).Acronyms are as follows B cells (B), Cytotoxic T cell (CTC), Dendritic cells (DC), Macrophages (MAC), Neutrophils (NEUT), Pan-T cells (Pan-T), Stromal cells (SC), T helper cells (THC), T regulatory cells (Treg). ns indicates no significance.

By a tiered classification approach, we first quantified the frequencies of tumor, immune and stromal cell subsets on a per-patient basis (**Figure 2A**). No significant differences were observed between these cellular subsets when comparing patients based on recurrence status. These results were compared to TIL grade scores (30) provided by pathologists on a serial section H&E slide which also showed no difference between the two response groups (**Supplemental Figure 3**). We then further resolved the immune cell subset into 8 phenotypic cell classes (B cells, helper T cells, cytotoxic T cells, T regulatory cells, neutrophils, monocytes, macrophages and dendritic cells) and quantified each immune cell subset as a fraction of total immune cells (**Figure 2B**). Among the 8 phenotypic cell classes assessed, no significant differences in cell counts were observed between any of the classes based on the recurrence response in patients following anti-PD1 therapy. Next, we quantified TIL subsets by their functional expression of PD1 (**Figure 2C**). Expectedly, PD1 expression was detected in a small but variable fraction of the TIL populations, however PD1 expression did not differentiate patients based on recurrence status. Overall quantification at the per-patient level of tumor, stromal, immune classes and PD1-expressing TIL subsets were insufficient in differentiating patients by recurrence outcome.

### Macro-architecture assessment of the tumor core region using cellular communities

**Figure 3:**
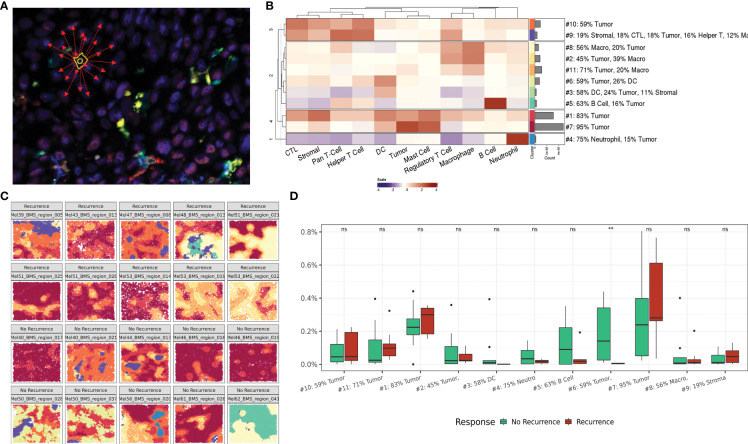
Regional interactions within cellular communities. **(A)** Representative illustration of cellular community definition. Each cell is defined by its 75 nearest neighbors. **(B)** Heatmap depicting cellular composition of 11 community clusters and relative frequency of each community cluster across the dataset. **(C)** Representative FOVs illustrating macroarchitecture of community clusters in recurrence and no recurrence cohorts. Community colors correlate with the heat map groups in panel **(B, D)** Box plot comparing community cluster frequencies between patients based on recurrence status. ns indicates no significance, ** indicates p value <0.01.

To spatially position single cell quantification within the tumor core region of the metastatic LN, we next evaluated cells within their surrounding cellular community. This data-driven approach was first described by Schurch et al. and individually annotates each cell based on a pre-defined number (n) of surrounding cells (nearest neighbors) (14). This n-cell composite is then computed for every cell across all FOVs for the entire patient cohort and combined to enable unsupervised clustering of a pre-defined number of community clusters (k) (**Figure 3A**). The resulting community subtypes can then be defined by their most enriched phenotypic cell class(es). For our study, we assessed communities of varying n size (5-250 neighbors) and k number of community clusters (5–50). A neighborhood size of 75 neighbors with 11 community clusters resulted in optimal clusters representative of macro-architecture of the tumor core.

The resulting community clusters were further defined by their most enriched (>10%) phenotypic cell class(es). A heat map showing the relative contribution of each cell class to each community subtype and the relative frequency of each community subtype demonstrates the intrinsic cellular heterogeneity that contributes to each community cluster and the distribution across the patient dataset (**Figure 3B**). Expectedly, two communities encompassing 66% of all communities were predominantly occupied by tumor cells (95% and 83% tumor respectively) reflective of the tumor core. Visualization of the community clusters projected back on the single cell resolved tumor core FOVs provides a macroarchitecture construct reflective of the regional structures within the tumor core as well as the diversity across patients and clinical outcomes (**Figure 3C**).

Comparing the frequency of community clusters between patients stratified on recurrence status, community #6 was significantly enriched in patients who did not experience recurrence following immunotherapy (**Figure 3D**). Community 6 was comprised of primarily tumor (59%) and DCs (26%) suggesting a role for active antigen presentation within the tumor core to mediate successful anti-tumor immune responses in the context of immunotherapy. Cellular communities provide a regional overview of the tumor-immune climate that spans the patient cohort and provides a framework for assessing regional differences between cellularly diverse tumor cores.

### Phenotypic and spatial paracrine interactions within cell-centered neighborhoods

**Figure 4:**
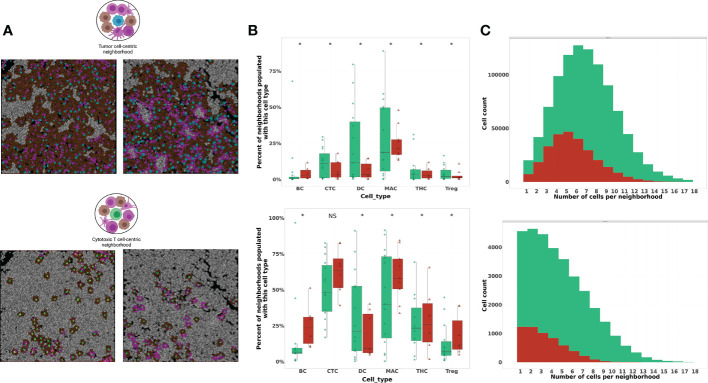
Phenotypic composition of cell-centered neighborhoods differs based on index cell and clinical recurrence status. **(A)** Illustration and visualization of all TCCN and CTCN with at least one immune cell (TCCN) or tumor cell (CTCN) present in tumor FOVs. In TCCN, index (center) tumor cell is indicated in blue, in CTCN index CTL is indicated in green, tumor cell neighbors are brown, immune cell neighbors are magenta. (TCCN n=983,823 and CTCN n=37,402) **(B)** Box plot comparing neighborhood frequencies of immune composition between patients based on recurrence status. Distribution of patients displayed on box plot as green circles (no recurrence) and red triangles (recurrence). **(C)** Distribution of tumor cell counts in TCCN and CTCN in patient cohorts. Acronyms are as follows B cells (BC), Cytotoxic T cell (CTC), Dendritic cells (DC), Macrophages (MAC), T helper cells (THC), T regulatory cells (Treg). NS indicates no significance, * indicates p value <0.01.

To investigate the paracrine interactions occurring between individual cells and their nearest spatial neighbors, we next applied a cell-centric neighborhood approach. We previously established the biologic value of tumor-centric cellular neighborhoods through identification of immune signatures in distinct regions of the metastatic LN (20). In this study, we applied the same cell-centric neighborhood approach to two index cell types: tumor cells (TC) and CTLs (CT) (**Figure 4A**). Neighborhood composition was compared between patient cohorts by evaluating the percent of neighborhoods populated with immune cell phenotypes classified on a per-patient basis. In tumor-centric cellular neighborhoods (TCCN), patients who did not experience recurrence had a higher proportion of TCCN populated with most immune cell subsets (CTL, DC, helper T cells and Tregs) compared to patients who did not recur. Notably, B cells and macrophages had an increased representation in neighborhoods of patients who experienced recurrence (**Figure 4B**).

Next, we considered neighborhood composition when a CTL was the center of the cellular neighborhood (CTCN). Unlike in TCCNs, CTCN had an increased presence of immune cell subsets including B cells, CTLs, Tregs, helper T cells and macrophages in neighborhoods derived from patients who experienced recurrence compared to those who did not. In patients that did not experience recurrence only DCs were enriched in CTCN. This finding suggests that CTLs in responders may be preferentially surrounded by antigen-presenting DCs while non-responders were more frequently surrounded by other immune cell subsets and may have fewer opportunities to engage with the intended target of tumor cells. To determine the tumor cell contribution to TCCN and CTCNs, the frequency of neighborhoods was plotted against the number of tumor cells in each neighborhood type by response cohort (**Figure 4C**). In TCCN, the average number of tumor cells in a neighborhood was 7 for patients that did not recur compared to an average of 5 tumor cells per TCCN in patients that experienced recurrence. In the CTCN setting, the average number of tumor cells per neighborhood was comparable between patient cohorts.

**Figure 5:**
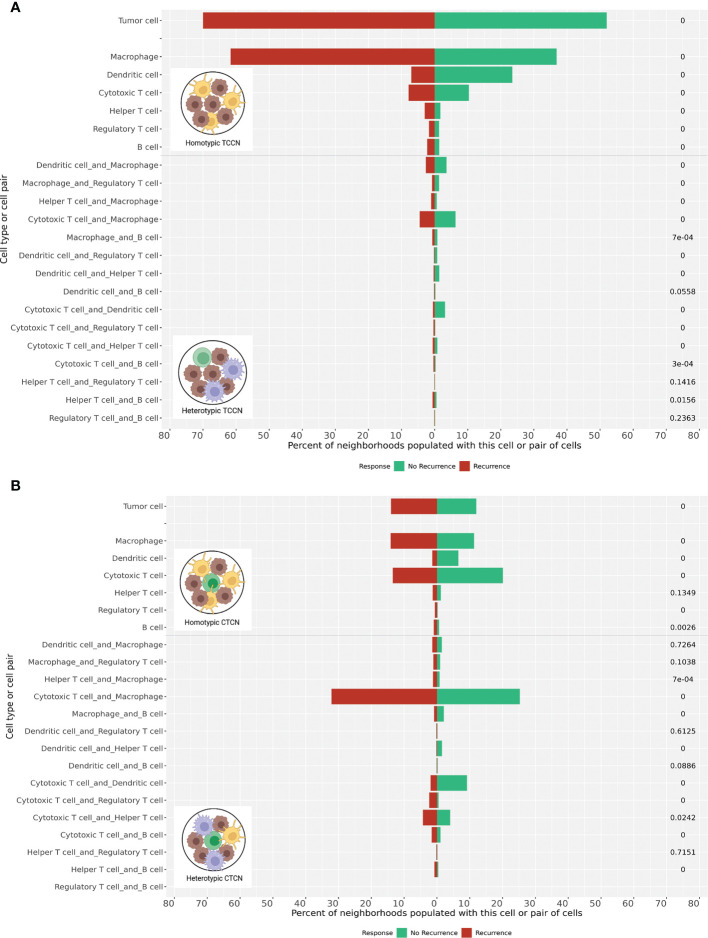
Homotypic and heterotypic cellular interactions within TCCN and CTCN. **(A)** Frequency of tumor-only and immune-populated neighborhoods were computed in TCCN of patients that recurred vs those that did not. Frequency of immune-populated TCCNs were further categorized based on presence of a single immune cell subset (homotypic) or two distinct immune cell classes (heterotypic) within the neighborhood. **(B)** Frequency of tumor only, immune homotypic and immune heterotypic neighborhoods were similarly assessed in CTCN of patients that experienced recurrence vs those that did not. p values indicated on right side of each bar. 0 represents p value< 0.00001.

We then evaluated the paracrine, pairwise cellular interactions present within these distinct cell-centric neighborhoods (**Figure 5**). Building from previous work evaluating pairwise interactions in size-defined neighborhoods (14), we adapted the nomenclature of homotypic and heterotypic neighborhoods for our cell-centric neighborhood definition to represent neighborhoods comprised of a single immune cell class (homotypic) or a combination of at least two unique immune cell classes beyond the index cell (heterotypic). In patients that experienced recurrence, homotypic TCCN and CTCN neighborhoods comprised of either macrophages or tumor cells were dominant, while DCs and CTLs homotypic neighborhoods were most frequent in patients that did not experience recurrence.

We also considered heterotypic immune cell pairings within the TCCN and CTCN finding distinct pairings between the two response groups. Most notably, the macrophage-CTL pairing was the most frequent in the CTCNs of patients that experienced recurrence while the DC-CTL pairing was most frequent in both CTCN and TCCNs of patients that did not recur. Taken together, these pairwise interactions further delineate distinct paracrine immune interactions signatures across patients with similar clinical responses to anti-PD1 therapy and suggest a role for lymphocyte-myeloid interactions in the context of the TIME.

### Evaluating the PD1-PDL1 functional axis in the context of the TCCN

**Figure 6:**
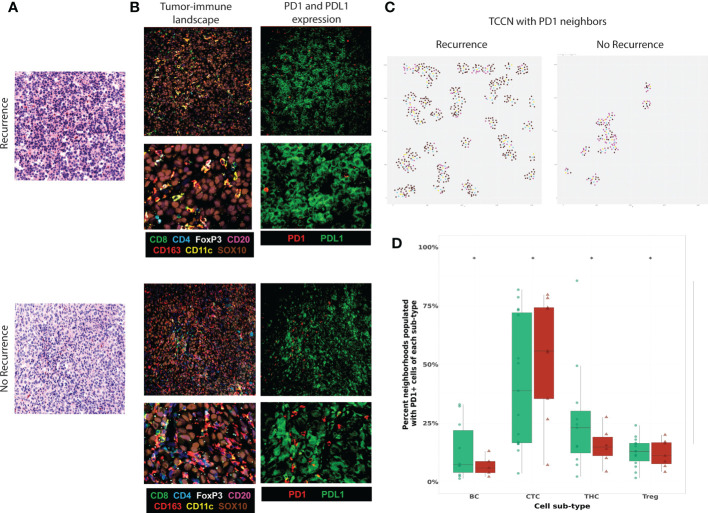
Functional expression of PD1-PDL1 contextualized in the TCCN of the tumor-immune climate. **(A)** Virtual H&E of representative tumor FOVs. **(B)** MxIF overlay details tumor-immune landscape (TILs, myeloid cells, tumor cells) and PD1 and PDL1 expression in the same FOVs. **(C)** R plots identify spatial TCCN with at least one PD1+ neighbor (index tumor cell: teal, PD1+ cell: yellow, immune cell: purple, tumor cell: brown). **(D)** Box plot comparing frequency PD1+ TIL subsets as neighbors in TCCN between patients based on recurrence status. Distribution of patients displayed on box plot as green circles (no recurrence) and red triangles (recurrence). Acronyms are as follows B cells (BC), Cytotoxic T cell (CTC), T helper cells (THC), T regulatory cells (Treg). * indicates p value <0.01.

To evaluate the PD1-PDL1 signaling axis in the context of neighborhoods, we next considered PD1 and PDL1 expressing cells in the TIME. Tumor-expressing PDL1 was observed in a subset of tumor FOVs (**Figure 6A**) derived from patients irrespective of recurrence status but was only expressed in a minority of cases and therefore not considered for quantitative analysis. Visualization of representative FOVs indicates PD1 expressing immune cells were present in PDL1 expressing tumor areas (**Figure 6B**) and TCCNs comprised of at least 1 PD1-expressing TIL were projected on the representative FOV map (**Figure 6C**). We then quantitatively considered PD1 expressing TIL neighbors in tumor-centric neighborhoods (**Figure 6D**). PD1 expressing B cells, T helper cells and Tregs showed an enriched frequency in TCCN of patients that did not experience recurrence. Unexpectedly, PD1 expressing CTLs were enriched in TCCNs of patients that experienced recurrence. This finding suggests other TIL subsets beyond CTLs may be responsible for mediating anti-PD1 mechanistic effects in the tumor core setting and further supports the underlying mechanism that PD1 expression or TIL presence alone is insufficient in predicting clinical outcomes to anti-PD1 therapy.

## Discussion

**Figure 7:**
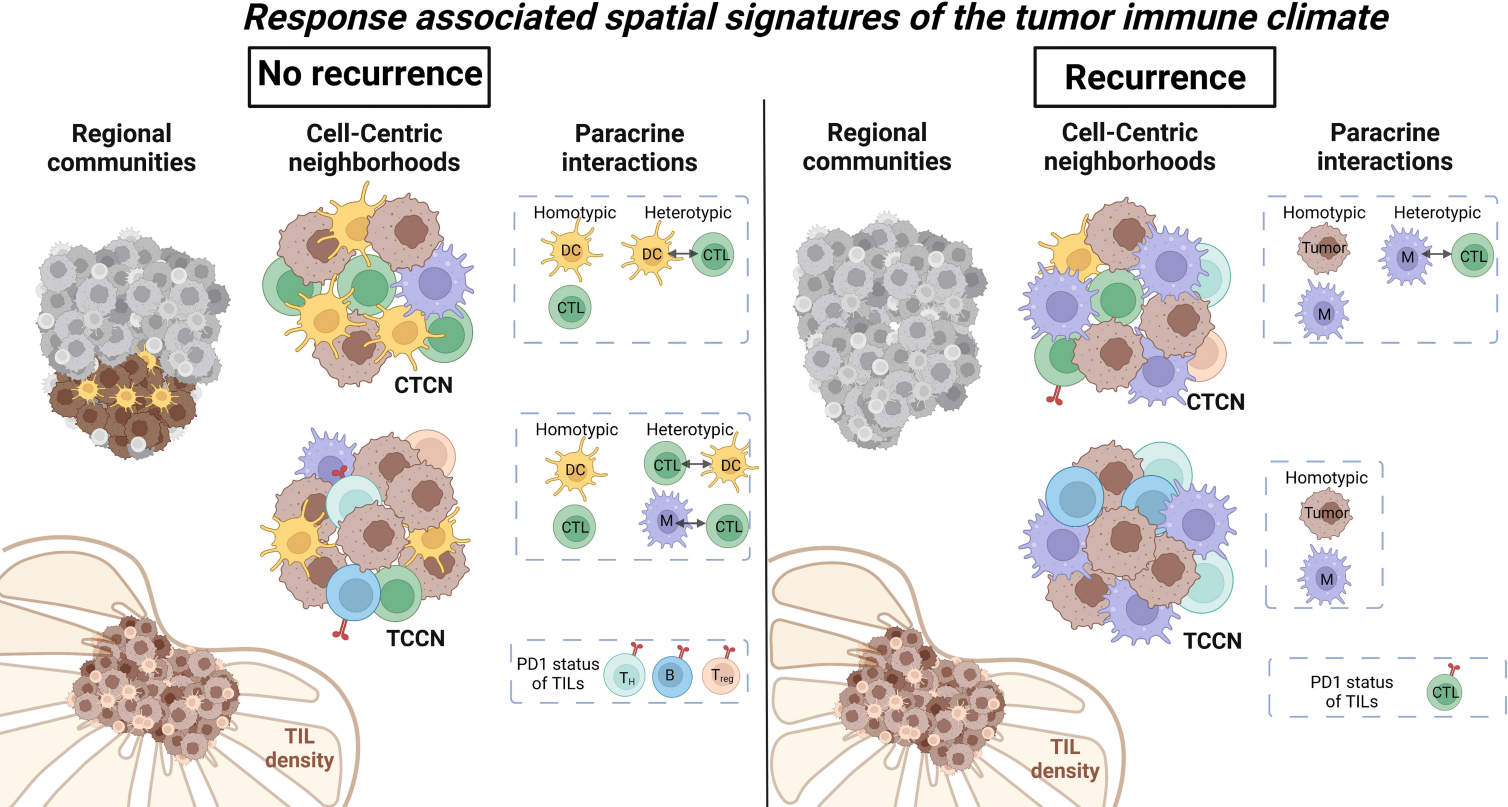
Summary of regional and paracrine level differences within metastatic lymph nodes based on recurrence status. Regional cellular communities identified a cluster comprised of primarily DCs and tumor cells to be enriched in patients that did not experience recurrence. Paracrine tumor-centric neighborhoods showed increased immune cell diversity while CTL-centric neighborhoods were enriched for DCs in patients that did not recur. Immune cell interactions within neighborhoods favored DC and CTL in patients that did not recur and macrophages and CTL in patients that did experience recurrence. Illustration was created using Biorender.

Although previous work demonstrates TIL presence is prognostic in various solid tumor settings (1–5), our data suggests that TIL diversity and paracrine inter-cellular immune interactions at the level of the TIME may be more relevant to the outcomes of PD1 directed immunotherapy in the setting of metastatic melanoma. In our patient level analysis, cell number quantification and conventional TIL scoring by H&E assessment of the tumor core found that cellular subset frequencies alone were insufficient in differentiating patients based on recurrence risk. By combining cell quantification with its surrounding spatial context, cellular communities identified an enriched community cluster comprised of primarily tumor cells and DCs in patients that did not experience melanoma recurrence. This predilection for DC interactions was further supported when assessing paracrine interactions in cellular neighborhoods centralized around either CTLs (CTCN) or tumor cells (TCCN). Patients with a favorable outcome showed an increase in DC-CTL interactions in both CTCN and TCCN while patients that experienced recurrence showed an enrichment for B cells and macrophages in comparable neighborhoods. Overall, these findings suggest that myeloid cells may play a critical role in modulating the effector-target interaction between CTLs and tumor cells in the metastatic LN setting (**Figure 7**).

DCs are the central antigen presenting cells with the capacity to prime and polarize T cells for antigen-specific effector functions (31). Within the TIME, mature DCs are required to activate tumor-specific CTLs for tumor cell lysis *via* granzyme release (32). However, accumulating evidence suggests that DCs in the TIME may become compromised in their antigen presenting capacity (9). Additional studies have shown an overall increase in other myeloid cell subsets in the TIME including tumor-associated macrophages and myeloid-derived suppressor cells fostering an immunosuppressive microenvironment (29). Our novel findings add to this emerging tumor-immune landscape to suggest that the spatial positioning of specific myeloid cell subsets in relation to tumor and CTLs may be critical for differentiating favorable immune responses to anti-PD1 therapy. Notably, overall abundance frequencies of myeloid cell subsets did not differentiate patients based on recurrence status following anti-PD1 therapy. However, in patients that did not experience nodal recurrence, we observed TCCNs and CTCNs were preferentially occupied by DCs over macrophages, and the pairwise interaction between DCs and CTLs in tumor-centric neighborhoods was more pronounced when compared to patients that experienced recurrence. These findings highlight the value of evaluating tumor-immune interactions within the spatial context of a given TIME suggesting a non-binary model governing the outcome of CTL-tumor cell interactions. The presence of a modulator cell influencing the outcome of the CTL-tumor cell contact also creates an opportunity for new therapeutic target development.

The PD1-PDL1 signaling axis continues to be a promising immunotherapy target in multiple solid tumors (33). The role of PD1 in promoting tolerogenic responses in T cells is well established (34). Accumulating evidence indicates immune checkpoint inhibitor therapies targeting PD1 can reactivate T cells and improve host immune responses (35). A recent study conducted by Johnson et al. utilized serial tissue sections and low-plex spatial imaging to identify predictive biomarker signatures in patients with melanoma undergoing anti-PD1 therapy. The interaction scores between PD-1/PD-L1 and IDO/HLA-II correlated with improved progression-free and overall survival (36). To see whether signatures indicative of these favorable responses could be identified at the resolution of paracrine interactions in advance of PD1 therapy, we evaluated TIL expression of PD1 and tumor cell expression of PDL1 in TCCNs. Representative FOVs with PDL1-expressing tumor cells illustrated regions with PD1 infiltration but quantitative assessment of PD1-expressing TIL subsets showed a diversity in TIL subsets associating with recurrence outcomes. Overall, this data suggests exploration of the PD1 mechanism of action in TILs beyond CTLs in the tumor setting is warranted.

Lymph nodes are critical secondary lymphoid organs and centralize the immune cells required for mounting virtually every host immune response (37). As such, the cellular architecture of the LN is highly organized for its primary purpose of immune cell differentiation, maturation and effector functions (37). Under homeostatic conditions these distinct architectural structures create functional multi-cellular units of B-cell enriched LN follicles and T cell-DC enriched LN cortex regions. The spatial arrangements of these tissues are directly related to their cellular function. In this study, we demonstrate that the tumor-immune climate within the LN may also be spatially regulated. Within the tumor core, TCCNs derived from patients that did not experience recurrence showed a greater diversity in immune cell composition and immune cell interactions including DC interactions with CTLs, macrophages and Tregs. In contrast, TCCNs derived from patients that experienced recurrence were occupied primarily by macrophages and B cells with the macrophage-CTL interaction being the most pronounced. These distinct paracrine immune signatures illustrate the diversity of cellular interactions that are promoted in otherwise comparable TIME based on immune cell quantification alone. As multiplex bioimaging platforms continue to evolve, cross-disciplinary applications also expand. Recent work conducted by Gong et al. applied pharmacology-based computational modeling to study the spatial heterogeneity at the level of tissue organs and patients in solid tumors including non-small cell lung cancer. Combining predictive modeling with high resolution multiplexed bioimaging platforms provides a new frontier for biomarker development and stratifying patients to clinical regimens (38). Ongoing studies will consider the functional status of these different immune cell subsets through expression of maturation, activation and differentiation markers as well as expanded evaluation of distinct histologies within the LN landscape. In evaluating the functional diversity of immune cell subsets in the context of the TIME we hope to further resolve the extensive heterogeneity in tumor-immune landscapes to derive predictive immune signatures related to immunotherapy responses.

Our novel study has several strengths. To our knowledge, this is the first study to apply multiplex imaging technologies to interrogate the phenotypic diversity and spatial dynamics of TIL subsets in the metastatic tumor setting. High accuracy single cell segmentation and a 10-class phenotypic classification model was achieved by leveraging our 45-antibody panel design and a machine-based modeling approach. Furthermore, we adapted existing spatial tools to evaluate the single-cell TIME at various levels of resolution (patient, regional, paracrine, pairwise and single-cell functional). Our novel observations that the myeloid compartment may play a more direct role in modulating the CTL-tumor interaction in the LN TIME highlights the need for studying cellular network interactions beyond pairwise associations to contextualize immunotherapy in this dynamic tissue landscape.

We also recognize limitations to our approach. While we assessed 416 pathologist-classified tumor core FOVs across a cohort of 25 patients, our dataset remains exploratory. We chose to focus on the tumor-enriched areas of the metastatic LN to better represent conventional TIL evaluations in primary tumor setting, however, this significantly reduces the number of immune cell observations per FOV and future studies will be needed to validate these findings in similar tumor regions and other histopathologically relevant areas of the TIME. Similar to previous reports (39), 9 patients (36%) in this cohort experienced melanoma recurrence following anti-PD1 therapy in less than 12 months. Future studies will be required to validate our findings in larger cohorts. While 10 phenotypes were classified with acceptable accuracy, future studies will continue to diversify immune cell subsets and the functional status of these subsets by leveraging additional markers in the panel design.

The importance of our study is that it offers a more detailed evaluation of TILs and spatial-immune signatures in the metastatic setting and informs recurrence risk following immunotherapy. Beyond the known associations between immune-infiltrated tumors and favorable responses to immune checkpoint inhibitors (21), our findings highlight the diversity of cellular interactions that contribute to heterogeneous responses to immunotherapy in addition to the intended effector-target mechanism of action. Further exploration of the myeloid cell contribution to this interaction, the maturation and functional polarization of these cells as well as the spatial arrangement of immune cells in the overarching tissue context will provide critical insights to improve the current immunotherapy landscape.

## Data availability statement

The original contributions presented in the study are included in the article/**Supplementary Material**. Further inquiries can be directed to the corresponding author.

## Ethics statement

The studies involving human participants were reviewed and approved by Mayo Clinic Institutional Review Board. Written informed consent for participation was not required for this retrospective study in accordance with the national legislation and the institutional requirements.

## Author contributions

RLM, AAL, RMM, BJC and SM contributed to the study conception and design. RLM and CA performed the experiments and data acquisition. RMM and ZF developed methodology and data curation. RG and BT served as consulting pathologists for antibody validation and tissue scoring. BD and TD conducted clinical record review and contributed to patient selection. JS, BJC and SNM supervised the study and secured funding support. RLM, AAL, RMM, TD and SM prepared the initial manuscript draft. All authors contributed to the article and approved the submitted version.

## Funding

Support for this work was provided by Bristol-Myers Squibb (CA209-7XD), Mayo Clinic Cancer Center (CA015083) and Mayo Foundation.

## Acknowledgments

The authors would like to thank the Pathology Research Core for providing tissue sectioning and H&E staining services. Figure illustrations were created using Biorender.com. The authors would like to thank the physicians of the Mayo Clinic Melanoma Oncology division who treated the patients included in this study. We also wish to extend our gratitude to the patients and their families for their commitment to scientific advances in metastatic melanoma.

## Conflict of interest

The authors declare that this study received funding from Bristol Myers Squibb. BJC is an employee and stockholder of Bristol Myers Squibb, and contributed to the study design and review of the manuscript.

The remaining authors declare that the research was conducted in the absence of any commercial or financial relationships that could be construed as a potential conflict of interest.

## Publisher’s note

All claims expressed in this article are solely those of the authors and do not necessarily represent those of their affiliated organizations, or those of the publisher, the editors and the reviewers. Any product that may be evaluated in this article, or claim that may be made by its manufacturer, is not guaranteed or endorsed by the publisher.
