# Supplementary material for: Quantitative spatial evaluation of tumor-immune interactions in the immunotherapy setting of metastatic melanoma lymph nodes
Source: Front Immunol. 2022 Dec 5;13:1024039. doi: 10.3389/fimmu.2022.1024039 (PMC9760971; doi:10.3389/fimmu.2022.1024039)
Supplement: Supplementary file 1 [file DataSheet_1.pdf]

Supplemental Materials

Supplemental Table 1: Patient Demographics

**PATIENT CHARACTERISTICS**				
	Relapse < 1 year of NIVO start		Relapse > 1 year of NIVO or no relapse	
N	9		16	
Mean Age at initial diagnosis (SD)	61.1 (16.1)		58.4 (13.2)	
Mean follow-up (days) after NIVO (SD)	683.3 (322.5)		906.4 (321.5)	
Average time (days) from sample to start of adjuvant therapy (SD)	38.3 (19.1)		45.4 (19.6)	
	N	%	N	%
Female	3	33.3%	6	37.5%
Prior non-melanoma skin cancer diagnosis	4	44.4%	3	18.8%
Prior radiation	1	11.1%	1	5.3%
Tumor characteristics at initial diagnosis				
Mean Breslow thickness, mm (SD)	2.5 (2.0)		3.5 (2.3)	
Breslow thickness not recorded	0	0.0%	5*	31.3%
Ulceration present	1	11.1%	4	25.0%
Ulceration not recorded	1	11.1%	7	43.8%
Average Mitotic rate, per mm^2 (SD)	2.5 (2.0)		5.3 (4.1)	
Mitotic Rate not recorded	1	11.1%	6	37.5%
Detected Mutations				
BRAF V600E	4	44.4%	5	26.3%
OTHER	1	11.1%	4	21.1%
None identified or testing not done	4	44.4%	10	52.6%
Study Sample collected at initial diagnosis or recurrence				
Initial Diagnosis	3	33.3%	11	68.8%
Recurrence	6	66.7%	5	31.3%

* Four patients with unknown primary and one patient with initial pathology unavailable

Supplemental Table 2: Antibody Panel Design

Conjugate	Antibody Name	Vendor	Cat No
Cy 2	CD15	abcam	ab212391
	ASMA	abcam	ab240654
Cy 3	CD4	abcam	ab181724
	CD8	Novus Biologicals	2PABX210601
	CD31	abcam	ab222431
	CD27	abcam	ab192336
	CD56	abcam	ab215981
	CD206	abcam	ab64693
	HLA-I	abcam	ab70328
	CD163	Bio-rad	MCA1853
	CD20	abcam	ab166865
	CD11C	abcam	ab216655
	CD21	abcam	ab240987
	TCF7	Cell Signaling	2203BF
	PANCK	Sigma	CS992
	Ki67	abcam	ab197547
	SOX10	abcam	ab239751
	KAPPA	abcam	ab232152
	CD38	abcam	ab204940
	MPO	abcam	ab221847
	CD19	abcam	ab237772
	Tryptase	Biolegend	369402
	GP100	abcam	ab787
Cy 5	NaKATPase	abcam	ab76020
	FOXP3	abcam	ab96048
	PDL1	abcam	ab221612
	CD3	abcam	ab17143
	GZB	abcam	ab219803
	CD28	R&D Systems	AF-342-PB
	CD86	Cell Signaling	91882BF
	CD45	abcam	ab8216
	CASP3	Cell Signaling	96618F
	CD16	abcam	ab215977
	CTLA4	BioSB	BSB2885
	CD11b	abcam	ab238794
	LAG3	abcam	ab227579
	S6	Santa Cruz	SC-74459 AF647
	HLAII	Santa Cruz	SC-53302
	CD68	Epredia	MS-397P
	CD14	abcam	ab214438
	MART1	abcam	ab212366
	PD1	abcam	ab186928
	B2M	abcam	ab237032
	LAMBDA	abcam	ab196673
	ANXV	Biolegend	640901

Supplemental Table 3: Phenotypic cell classification model. For manual expert annotations in QuPath the following immune, tumor and stroma cell subsets were differentiated by leveraging co-expression of the antibodies in the MxIF panel. Expert annotations were used as the training dataset for the phenotypic classification model described in the Methods.

**Immune**								**Tumor**	**Stroma**
Cytotoxic T cells	B cell	Tregs	Macrophage	Dendritic cells	Helper T cells	Neutrophil	Monocyte	Tumor	Stroma
**CD8**									
CD3	**CD20**								
	CD19	**FoxP3**			No FoxP3				
	CD21	CD4	**CD163**		No CD11c				
		CD3	CD14	**CD11c**	No CD163				
			CD16	CD14	**CD4**		No CD11c		
			HLAII	CD16	CD3	**MPO**	No CD163		
				HLAII		CD15	**CD16**		
							CD14	**SOX10**	
								gp100	**aSMA**
								MART1	

Supplemental Figure 1: Virtual H&E maps of each lymph node tissue sample (n=25 cases). Pathologist-annotated tumor core regions used for analysis are indicated by red squares (n=416 FOVs).

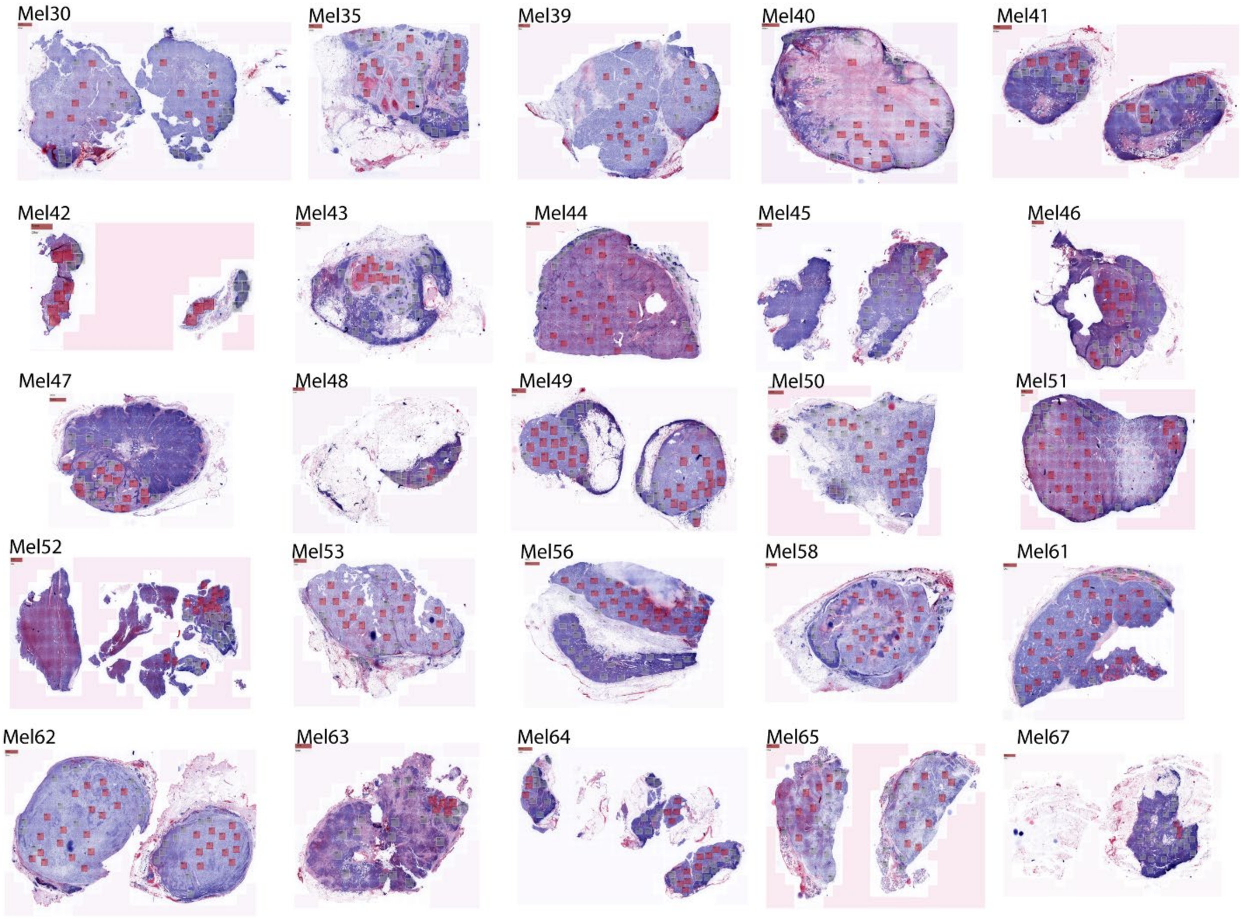

Supplemental Figure 2: Neighborhood size derivation. To determine optimal neighborhood size for the current histology (tumor FOVs in melanoma lymph node) all neighborhoods possessing at least 1 neighbor in addition to the center index cell were plotted from each FOV (line) against radius size in pixels ranging from 0-200 pixels (A). For the current tissue histology, a neighborhood size of 57 pixels represented the 90th percentile of all neighborhoods and was used as the neighborhood size for all downstream analysis (B).

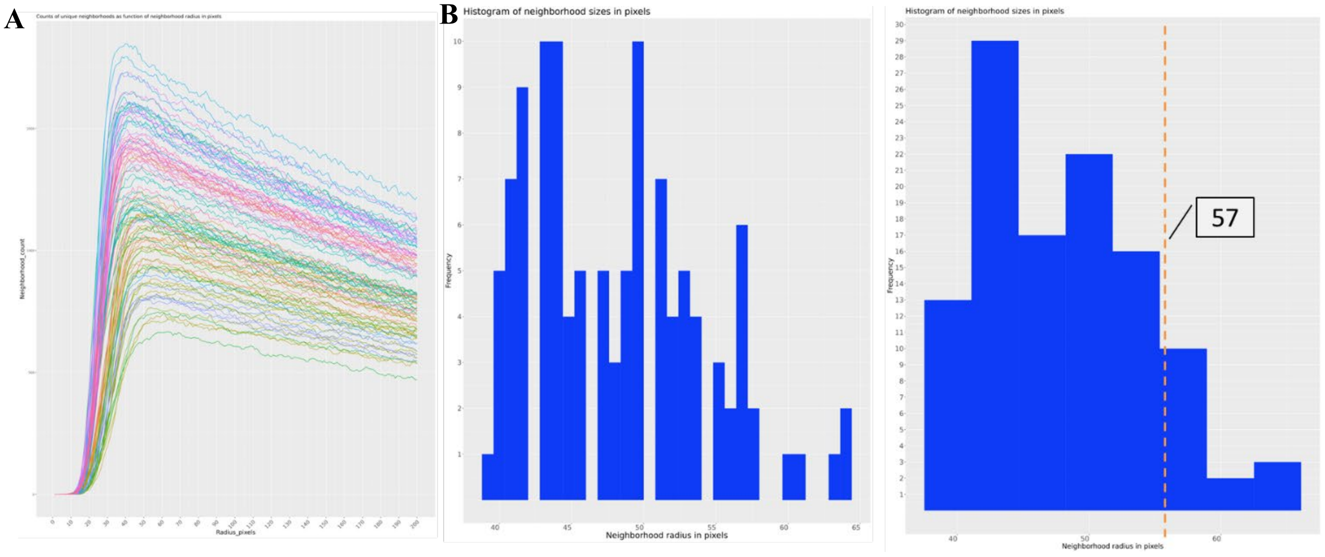

Supplemental Figure 3: TIL scoring of metastatic lymph nodes. H&E serial section of metastatic lymph node biopsy was scored according to TIL scoring criteria as previously described (ref 30). Average score was not significantly different between the response groups.

Recurrence		No Recurrence	
**Sample**	**Score**	**Sample**	**Score**
Mel30	1	Mel40	2
Mel35	1	Mel42	2
Mel39	2	Mel44	2
Mel41	1	Mel45	2
Mel43	2	Mel46	2
Mel47	1	Mel49	2
Mel48	2	Mel50	3
Mel51	2	Mel52	2
Mel57	3	Mel56	1
		Mel58	1
		Mel61	NA
		Mel62	NA
		Mel63	NA
		Mel64	NA
		Mel65	2
		Mel67	3
**Average**	1.666667		2
**Std Dev**	0.707107		0.603023
**T-test**	0.2723454		

Scoring criteria as defined by Ref 30.	
**0**	TILs absent
**1**	mild TIL infiltrate
**2**	moderate TIL infiltrate
**3**	marked TIL infiltrate

**Mel61-64 serial H&E not available for scoring*
